# Impact of flail chest injury on morbidity and outcome: ten years’ experience at a tertiary care hospital in a developing country

**DOI:** 10.1186/s12871-023-02185-y

**Published:** 2023-07-04

**Authors:** Khaled M. Alanwer, Ali Mohammed Refat, Essamedin M. Negm

**Affiliations:** 1 Anesthesia, Intensive Care, and Pain Management, Faculty of Medicine, Zagazig University, Zagazig, Egypt; 2 Cardiothoracic Department, Faculty of Medicine, Zagazig University, Zagazig, Egypt

**Keywords:** Flail chest, ICU, Morbidity, Mortality

## Abstract

**Background:**

One of the worst types of severe chest injuries seen by clinicians is flail chest. This study aims to measure the overall mortality rate among flail chest patients and then to correlate mortality with several demographic, pathologic, and management factors.

**Methodology:**

A retrospective observational study tracked a total of 376 flail chest patients admitted to the emergency intensive care unit (EICU) and surgical intensive care unit (SICU) at Zagazig University over 120 months. The main outcome measurement was overall mortality. The secondary outcomes were the association of age and sex, concomitant head injury, lung and cardiac contusions, the onset of mechanical ventilation (MV) and chest tubes insertion, the length of mechanical ventilation and ICU stay in days, injury severity score (ISS), associated surgeries, pneumonia, sepsis, the implication of standard fluid therapy and steroid therapy, and the systemic and regional analgesia, with the overall mortality rates.

**Results:**

The mortality rate was 19.9% overall. The shorter onset of MV and chest tube insertion, and the longer ICU, and hospital length of stay were noted in the mortality group compared with the survived group (*P*-value less than 0.05). Concomitant head injuries, associated surgeries, pneumonia, pneumothorax, sepsis, lung and myocardial contusion, standard fluid therapy, and steroid therapy were significantly correlated with mortality (*P*-value less than 0.05). MV had no statistically significant effect on mortality. Regional analgesia (58.8%) had a significantly higher survival rate than intravenous fentanyl infusion (41.2%). In multivariate analysis, sepsis, concomitant head injury, and high ISS were independent predictors for mortality [OR (95% CI) = 568.98 (19.49–16613.52), 6.86 (2.86–16.49), and 1.19 (1.09–1.30), respectively].

**Conclusion:**

The current report recorded mortality of 19.9% between flail chest injury patients. Sepsis, concomitant head injury, and higher ISS are the independent risk factors for mortality when associated with flail chest injury. Considering restricted fluid management strategy and regional analgesia may help better outcome for flail chest injury patients.

## Introduction

Severe blunt chest trauma remains one of the primary causes of morbidity and mortality in trauma patients of all ages. Flail chest is one of the deadliest kinds of traumatic chest injury, and it is certainly the most common major thoracic injury encountered by clinicians [1]. Flail chest is a condition defined as two or more contiguous rib fractures with two or more breaks per rib and has mortality estimates conservatively ranging from 9 to 20% [2, 3]. Such high mortality rates are attributed to a higher incidence of concomitant injuries and respiratory difficulties [2].

Flail chest injuries are estimated to affect 0.07% of hospitalized fracture rib patients [4], with 11.9% dying within 30 days [5]. According to the Trauma Audit and Research Network [6], more than 15,000 patients with rib fractures are hospitalized in the UK each year. In 2019, injury mortality rates in the Eastern Mediterranean area were estimated to be higher than worldwide rates [7].

Flail chest injuries according to abbreviated injury score (AIS) are graded as 3 or even more according the extent of injury and associated underlying organ damage. Injury severity score (ISS) is an anatomical scoring system which provides an overall score for patients with multiple injuries. Each injury allocated to one of six body regions is assigned an AIS score and the 3 most severely injured body regions have their score squared and added to produce the ISS score. ISS is globally recognized as one of injury scoring systems to predict outcome for polytrauma cases [8].

The gold standard for treating flail chest has traditionally been conservative non-operative care, which is defined as mechanical ventilation along with good pain management; nevertheless, mechanical ventilation for more than three weeks is frequently linked with various ventilation-related sequelae. Pneumonia occurs in 27 to 70% of flail chest patients who are not surgically treated, with a 51% mortality rate. Reports have found significant variations in mortality and morbidity among various healthcare facilities. The number of fractured ribs and the age of the patient both raise the risk of morbidity and mortality [9].

Sepsis complicating cases of polytrauma can gravely endanger prognosis, whether the source was an open or surgical wound or as a complication from mechanical ventilation. In this context, improper wound care, prolonged mechanical ventilation, or faulty antimicrobial regimen can eventually be detrimental [10].

Although intravenous analgesia is commonly used to relieve pain, it has a number of adverse effects [9]. There are currently insufficient data to demonstrate mortality reduction with regional blocks; however, the improved analgesia and decreased need for opioids offered by regional blocks are a meaningful endpoint in themselves [11]. Compared to other analgesic techniques, epidural analgesia offers better pain relief and improves the injured patient’s pulmonary function tests [12]. Also, various studies have shown the effectiveness of paravertebral block (PVB) as an effective analgesic option [13].

Therefore, a retrospective observational study was conducted using electronic records and patient-documented files from the Emergency Intensive Care Unit (EICU) and Surgical Intensive Care Unit (SICU) at Zagazig University. The primary goal of this study was to determine the overall mortality rate among flail chest patients admitted to the EICU and SICU and then to correlate that rate with a variety of demographic, pathologic, and management factors.

## Materials and methods

This retrospective observational study was carried out in the EICU and SICU at Zagazig University Hospitals. Zagazig University Hospitals are tertiary care facilities that serve and receive severe and complex polytraumatized patients from the governorates of Delta, Sinai, the Suez Canal, and the Red Sea. The EICU and SICU had 25 and 30 beds, respectively, during this study period. All patients with flail chest injuries who were admitted to ICU as a result of a road traffic accident between June 2011 and the end of May 2021, and who were eighteen years of age or older and had complete medical records during a ten-year period, were included in the study. Amongst 397 patients, 376 were included and 21 patients were excluded due to incomplete data, admission with a post cardiac arrest, or mortality within the first 24 h.

Flail chest injury diagnosis was confirmed radiologically by chest computed tomography after clinical suspicion. The main outcome measurement was overall mortality. The secondary outcomes were the association of demographic data, the incidence of concomitant head injury with abbreviated injury score (AIS) ≥ 3, onset and duration for the application of mechanical ventilation (MV) and chest tube insertion, ICU duration and total hospital stay, ISS, type of used analgesia, coincident or complicating pathologies, coincident myocardial injury; diagnosed by abnormal The electrocardiogram (ECG) and cardiac enzymes or by transesophageal echocardiography (TEE) (right or left ventricular systolic dysfunction, pericardial effusion with possible tamponade, ventricular septal defect, or suspected trauma-induced valvular abnormalities) [14], fluid management strategy, surgeries performed (head, abdominal exploration, thoracotomy, orthopedic, vascular, and tracheostomy), and steroid therapy with the overall mortality rates.

Restricted strategy was applied by restriction of IV fluids to less than 1,000 ml during resuscitation and 50 ml per hour thereafter [15]. Patients received standard fluid management strategy received 2 L of fluid as an initial bolus. Following the initial bolus, additional fluid was given as needed to maintain a SBP of 110 mmHg [16].

### Ethical approval

Under the approval by the research ethical committee of the Faculty of Medicine, Zagazig University (reference number ZU-IRB#: 9301-20-2-2022) with the consideration of the retrospective design of the study, patient informed consent was waived. The study was carried out in accordance with the principles of the Declaration of Helsinki and STROBE guidelines.

### Data analyses

The given data was collected through a thorough history, a simple clinical inspection, laboratory and radiological examinations, and conservative and operative management. The collected data was revised, coded, tabulated and introduced to a PC using Statistical package for Social Science (IBM Corp. Released 2017. IBM SPSS Statistics for Windows, Version 25.0. Armonk, NY: IBM Corp). Data was presented and suitable analysis was done according to the type of data obtained for each parameter. Kolmogorov–Smirnov was used to evaluate normal distribution of continuous data. Mean, Standard deviation (± SD), and range was used for parametric numerical data, while Median and Interquartile range (IQR) was used for non-parametric numerical data. Frequency and percentage of non-numerical data were used. Chi-Square test was used to examine the relationship between two qualitative variables. Mann Whitney Test (U test) was used to assess the statistical significance of the difference of a nonparametric variable between two study groups. Logistic regression was used to measure the relationship between the mortality variable and one or more independent variables.

## Results

**Fig. 1:**
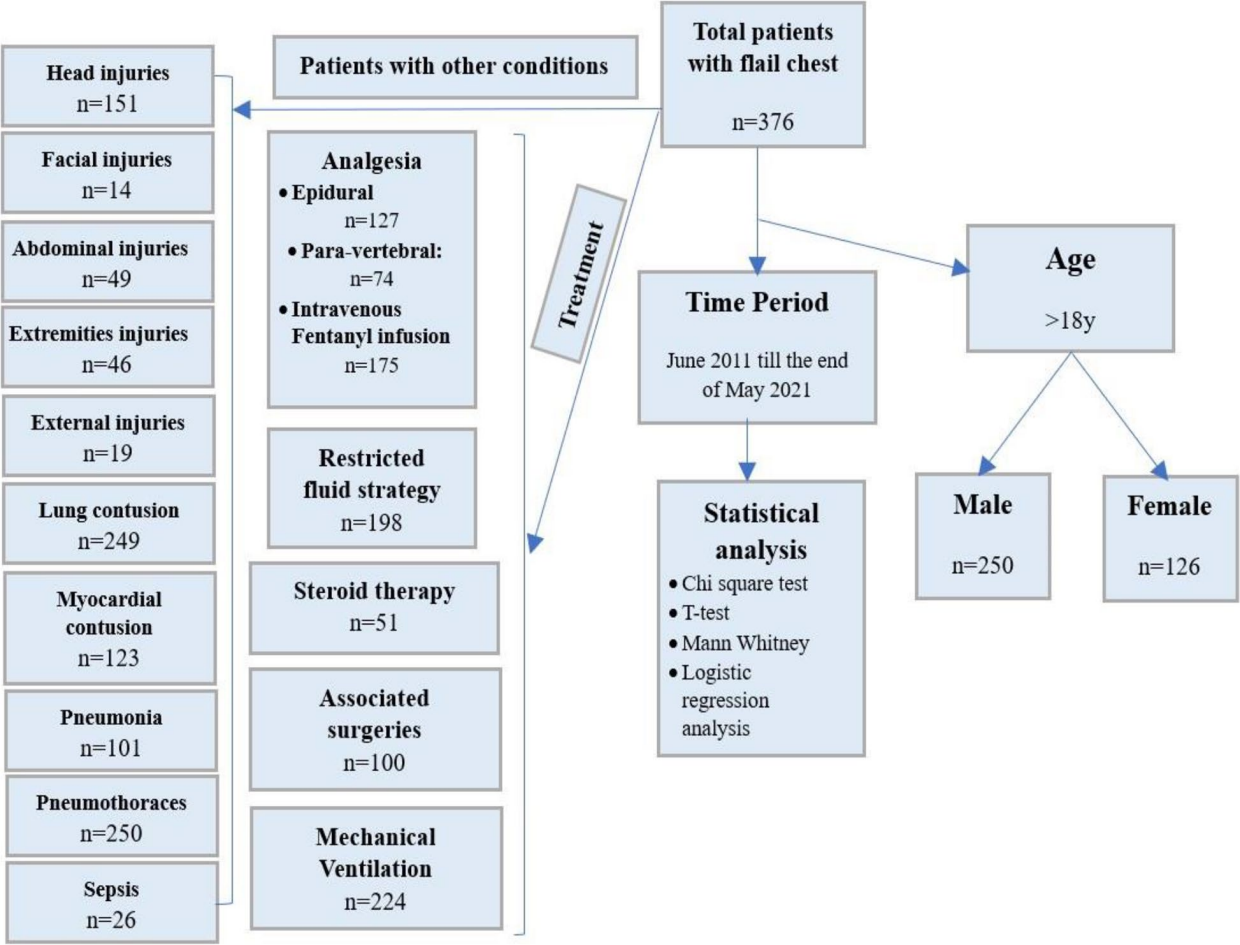
Flow chart describing the flail chest patients; demographic, associated, and management parameters

A total of *n* = 376 patients were admitted between June 2011 and the end of May 2021 with flail chest injuries. Figure 1 shows a flow chart describing the patient demographic data, and associated, and management parameters.

### Patient characteristics

#### Age distribution

**Fig. 2:**
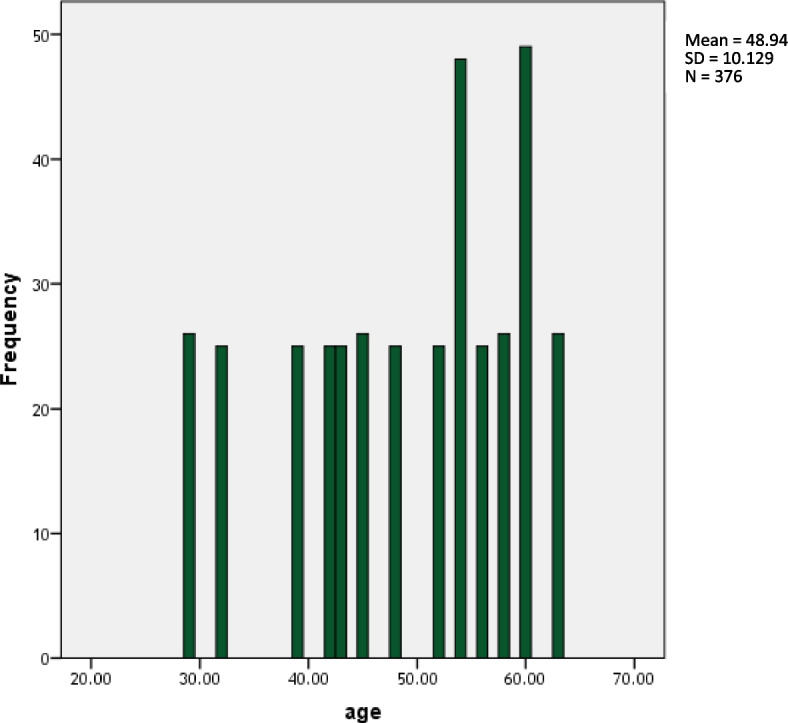
Age distribution among the studied group

The average age of the patients was 48.94 ± 10.12 years, with the youngest being 29 and the oldest being 63. The median range calculated from the data was found to be 52.0 (Fig. 2).

#### Gender distribution

Out of the total of 376 patients with a flail chest injury, 250 were males and 126 were female, representing 66.5% and 33.5% of the total studied group, respectively. Males represent about two-thirds of the studied group **(**Fig. 1).

#### Concomitant head injury distribution

fIn our study group, 151 casualties have had concomitant head injuries, representing 40.20% of the total number **(**Fig. 1)(Table 6).

### Onset and duration of mechanical ventilation and chest tube insertion

Due to the severity of the condition, 59.6% of patients (*n* = 224) needed MV; with a median onset of 2.0 with IQR of (1–4 days), which lasted for a median of 12.0 with IQR of (9–18 days) (Table 1). 66.5% of patients (Table 4) have developed pneumothoraces for which chest tubes were utilized. The chest tubes with a median onset of 1.0 with IQR of (1–3 days) with median duration of 6.0 with IQR of (4–9 days) were used for patients in order to stabilize their condition. The details are given in Table 1.

**Table 1:** Onset and duration of mechanical ventilation and chest tube insertion for the study cases

	MV onset (days)	MV duration (days)	Chest tube onset (days)	Chest tube duration (days)
**Median** (IQR)	2.0 (1–4)	12.0 (9–18)	1.0 (1–3)	6.0 (4–9)

#### ICU length of stay

A flail chest patient who had come to our hospital stayed for 22.69 ± 2.99 days, and the mean value of an ICU stay was 14.54 ± 2.93 days (Table 2).

**Table 2:** ICU and total hospital length of stay and ISS for the study cases

	ICU stay (days)	Hospital stay (days)	ISS
**Median** (IQR)	14.0 (10–21)	19.0 (14–45)	21.0(16–75)

*ISS *Injury Severity Score

### Treatments

#### Types of analgesia

In the current study, 46.5% of patients received continuous intravenous fentanyl infusion, whereas 19.7% of patients received continuous paravertebral analgesia, and 33.8% of patients received continuous thoracic epidural analgesia. The details of each are given in Table 3.

**Table 3:** Catheter-based regional analgesia and other techniques used to reduce pain-related complications

Analgesia	Number of patients	%
**Epidural**	127	33.8
**Para-vertebral**	74	19.7
**Systemic (IV Fentanyl)**	175	46.5
**Total**	376	100.0

#### Different clinical conditions and their treatments

Patients have developed different clinical conditions due to the severity of their injuries. Out of all the patients, 66.2% had lung contusion symptoms, 32.7% were diagnosed with myocardial contusion, 26.9% had pneumonia, and 6.9% had sepsis with a non-pulmonary origin. 52.7% of patients received treatment for these conditions using the restricted fluid strategy, and the remaining patients received standard fluid therapy. 13.6% of patients received steroid medication, while 26.6% underwent related operations. The details of each clinical condition and the applied treatment are given in Table 4.

**Table 4:** Clinical conditions and applied treatments of all flail chest injury patients

Clinical conditions		number of patients	%
**Lung contusion**	Yes	249	66.2
	No	127	33.8
**Myocardial contusion**	Yes	123	32.7
	No	253	67.2
**Pneumothorax**	Yes	250	66.5
	No	126	33.5
**Chest tube insertion**	Yes	250	66.5
	No	126	33.5
**Associated surgeries**	Yes	100	26.6
	No	276	73.4
**Pneumonia**	Yes	101	26.9
	No	275	73.1
**Sepsis**	Yes	26	6.9
	No	350	93.1
**Mechanical ventilation**	Applied	224	59.6
	Not applied	152	40.4
**Fluid**	Restricted	198	52.7
	Standard	178	47.3
**Steroid therapy**	Yes	51	13.6
	No	325	86.4
**Mortality (overall)**	Survived	301	80.1
	Died	75	19.9
	Total	376	100

### Relationship to the outcome

The overall mortality rate between our cases was 19.9% (Table 4). The 30-day mortality was 17.8%. In the elderly group, the mortality rate was much greater (Table 5). The shorter onset of MV and chest tube insertion, and the longer MV days, chest tube duration, ICU, and hospital length of stay were noted in the mortality group compared with the survived group (*P*-value less than 0.05) as shown in Table 5. Also, there was a statistically significant relation between ISS (injury severity score) and the mortality.

**Table 5:** Evaluation of the statistical significance of mortality based on clinical characteristics

Clinical characteristics	Died (*N* = 75)	Survived (*N* = 301)	Mann-Whitney U Test (^Z^_MWU_)	*P* value
	Median (IQR)	Median (IQR)		
Age (Years)	53.0 (42.0–66.0)	50.0 (40.0–58.0)	2.103	0.035*
Chest tube onset (hours)	24 (15.0–34.0)	29.5 (24.0–39.0)	9.206	0.000**
Chest tube duration (Days)	7.8 (4.4–12.2)	4.6 (3.7–6.5)	5.043	0.000**
MV onset (hours)	6.0 (2.0–32.0)	74 (45–96.0)	2.518	0.012*
MV duration (Days)	18.21 (10.9–26.0)	11.0 (6.0–18.0)	4.580	0.000**
ICU stay (Days)	29.33 (20.5–35.0)	9.8 (3.4–19.1)	9.855	0.000**
Hospital stay (Days)	41.8 (34.0- 48.9)	15.4 (9.3–26.0)	**11.044**	0.000**
ISS	43.0(38.0–54.0)	21.0(18.0–24.0)	11.769	0.000**

*Abbreviation*: *IQR *Interquartile range, *MV *Mechanical ventilation, *ICU *Intensive care unit, *ISS *Injury Severity Score; ** = statistically significant, *P < 0.05*

Furthermore, gender and MV use, had no statistical significance on mortality (Table 6).

**Table 6:** Study association and comparison in terms of outcome among given parameters

Details		Outcome		Total	X^2^	*P* values
		Survived	Died			
**Sex**						
** Male**	N	201	49	250	0.63	0.42
	%	66.7	65.4	66.5		
** Female**	N	100	26	126		
	%	33.3	34.6	33.5		
**Concomitant head injuries (AIS ≥ 3)**						
** Yes**	N	76	75	151	139.6	0.00**
	%	25.2	100.0	40.2		
** No**	N	225	0	225		
	%	74.8	0.0	59.8		
**Analgesia route**						
** Epidural**	N	120	7	127	26.2	0.00**
	%	39.8	9.3	33.8		
** Para-vertebral**	N	57	17	74		
	%	18.9	22.6	19.7		
** Systemic (IV fentanyl)**	N	124	51	175		
	%	41.2	68.0	46.5		
**Analgesia type**						
** Regional**	N	177	24	201	16.3	0.00**
	%	58.8	32.0	53.5		
** Systemic**	N	124	51	175		
	%	41.2	68.0	46.5		
**Lung contusion**						
** Yes**	N	177	72	249	37.1	0.00**
	%	58.8%	96.0%	66.2%		
** No**	N	124	3	127		
	%	41.2%	4.0%	33.8%		
**Myocardial contusion**						
** Yes**	N	63	54	117	73.1	0.00**
	%	20.9%	72.0%	31.1%		
** No**	N	238	21	259		
	%	79.1%	28.0%	68.9%		
**Pneumothorax**						
** Yes**	N	195	55	250	36.6	0.00**
	%	64.8	73.3	66.5		
** No**	N	106	20	126		
	%	35.2	26.7	33.5		
**Associated surgeries**						
** Yes**	N	30	70	100	211.3	0.00**
	%	9.9	93.3	26.6		
** No**	N	271	5	276		
	%	90.1	6.7	73.4		
**Pneumonia**						
** Yes**	N	51	50	101	75.5	0.00**
	%	16.9	66.7	26.9		
** No**	N	250	25	275		
	%	83.1	33.3	73.1		
**Sepsis**						
** Yes**	N	0	26	26	112.09	0.00**
	%	0.0	34.7	6.9		
** No**	N	301	49	350		
	%	100.0	65.3	93.1		
**Mechanical ventilation**						
** Applied**	N	174	50	224	1.95	0.16
	%	57.8	66.7	59.6		
** Not**	N	127	25	152		
	%	42.2	33.3	40.4		
**Chest tube insertion**						
** Needed**	N	195	55	250	36.6	0.00**
	%	64.8	73.3	66.5		
** Not**	N	106	20	126		
	%	35.2	26.7	33.5		
**Fluid**						
** Restricted**	N	174	24	198	16.0	0.00**
	%	57.8	32.0	52.7		
** Standard**	N	127	51	178		
	%	42.2	68.0	47.3		
**Steroid therapy**						
** Required**	N	20	31	51	61.6	0.00**
	%	6.7	41.4	13.6		
** Avoided**	N	281	44	325		
	%	93.3	58.6	86.4		
**Total**	N	301	75	376		
	%	100.0	100.0	100		

*N *Number of flail chest patients; ** = statistically significant, *P < 0.05*

**Fig. 3:**
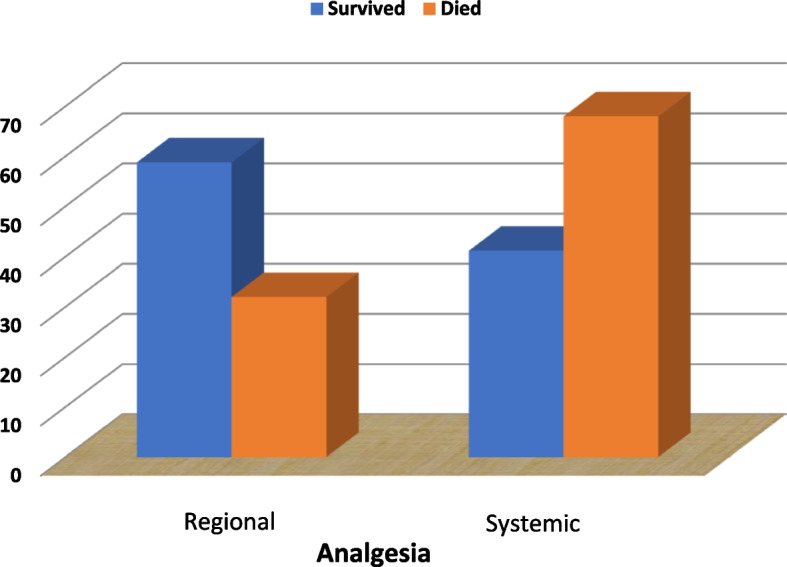
Relation between analgesia type and mortality among flail chest patients

Moreover, concomitant head injuries, lung and myocardial contusion, associated surgeries, pneumonia, sepsis, standard fluid therapy, and steroid therapy were significantly correlated with mortality (*P*-value less than 0.05). In our findings, regional analgesia had a significantly higher survival rate than intravenous fentanyl infusion (*P*-value less than 0.05) (Table 6) (Fig. 3).

Univariate and multivariate analysis were performed to investigate the possible predictive factors for mortality. In univariate analysis: cases with concomitant head injury, systemic fentanyl infusion, cases with lung contusion, myocardial contusion, pneumothorax, pneumonia, sepsis, and fluid restriction, chest tube insertion, steroid therapy, were correlated with increased risk of mortality. In multivariate analysis, using model adjusted for previously mentioned parameters, it was found that concomitant head injury, sepsis and high ISS score were independent predictors for mortality (Table 7).

**Table 7:** Logistic regression analysis for factors predicting mortality

Parameters	Univariate				Multivariate			
	*P*-value	Odds ratio (OR)	95%CI		*P*-value	Odds ratio (OR)	95%CI	
			Lower limit	Upper limit			Lower limit	Upper limit
**Concomitant head injury (AIS ≥ 3)**	**0.000**	5.516	3.719	8.180	**0.000**	**6.862**	**2.856**	**16.490**
**Analgesia: General/Regional**	**0.000**	3.033	1.773	5.188	0.999	36226116.4	0.000	.
**Lung contusion**	**0.000**	16.814	5.179	54.581	0.113	20.648	0.491	868.619
**Myocardial contusion**	**0.000**	9.714	5.464	17.272	0.999	0.000	0.000	.
**Pneumothorax**	**0.000**	16.584	5.108	53.840	0.108	0.062	0.002	1.848
**Associated Surgery**	**0.000**	19.232	10.370	35.667	0.163	0.242	0.033	1.779
**Pneumonia**	**0.000**	6.562	3.803	11.325	0.550	0.629	0.138	2.868
**Sepsis**	**0.000**	150.000	19.877	1131.967	**0.000**	**568.98**	**19.49**	**16613.52**
**Fluid restriction**	**0.000**	2.804	1.614	4.871	0.969	1.033	0.205	5.191
**Chest tube insertion**	**0.000**	29.456	13.891	62.461	0.083	7.061	0.776	64.246
**Steroid**	**0.000**	73.750	29.071	187.099	0.999	14989832.828	0.000	.
**ISS**	**0.000**	1.221	1.169	1.275	**0.000**	**1.188**	**1.086**	**1.300**
**Hospital stay**	**0.000**	1.006	1.005	1.008	**0.058**	1.014	0.890	1.028

*B* Regression coefficient, *S.E *Standard error, *CI *Confidence interval

## Discussion

Polytrauma patients frequently present with chest injuries. One of the most life-endangering injuries that indicate ICU admission is a flail chest injury. Mortalities related to flail chest injuries ensue from concomitant injuries as well as subsequent respiratory complications [17, 18]. Lung contusions [19] and lower respiratory tract infections and subsequent sepsis are the main respiratory complications contributing to mortality [17]. Currently, the main targets of management in intensive trauma units addressing respiratory challenges in such patients include different analgesic modalities as a part of or within general anesthesia, restricted fluid strategies, and steroid therapy. The success in restoring normal respiratory mechanics as well as the patients’ abilities to clear pulmonary secretions can delay or even amend the need for prolonged endotracheal intubation and/or mechanical ventilatory support, and hence the need for a tracheostomy. However, 30-day mortalities are still high in the global census for intensive care-enrolled flail chest casualties [18]. This study correlates different demographic characteristics, concomitant injuries, and management plans to the overall-day mortalities in EICU and SICU flail chest patients over 10 years. That is done in the hope of improving the current therapeutic plans.

The overall mortality rate noticed in our study was found to be 19.9%. That rate is relatively high when compared to published literature [7, 20, 21]. Road traffic injuries, caused on by high speeds and the lack of safe road infrastructure, continue to pose a major risk for the public health of the Eastern Mediterranean nations across all age groups. The late admission of complicated cases and the limited availability of rehabilitation services are explanations for the high mortality rate [7]. In the elderly group, the death rate was much greater. Elderly individuals are less likely to withstand the stressful condition, according to similar observations. Age-related alterations in breathing patterns are well recognized; for example, older adults who are at rest have faster respiratory rates and smaller tidal volumes, and their ventilatory response to hypoxia or hypercapnia is greatly reduced. Given this, it is logical to anticipate that posttraumatic respiratory failure, which worsens the prognosis, occurs more frequently in older individuals [22, 23]. Similarly, the shorter onset of MV and chest tube insertion was significantly higher in the mortality group.

Moreover, concomitant head injuries in this report were significantly correlated with mortality. Dehghan et al. reported similar findings, in which patients with concurrent acute head injuries had higher rates of ICU stay and ventilator support, as well as poorer outcomes [2]. Another systematic review and meta-analysis confirmed that concomitant head injury and age older than 65 years are particularly poor prognostic factors in patients with traumatic flail chest [24].

In this context, summing all squared AIS of body parts giving a final ISS score showed a direct correlation with mortality. This finding was consistent with several reports investigating the reliability of ISS score as an independent predictor for mortality in polytrauma patients [8, 25, 26].

Ventilatory support is usually indicated for patients with multiple extra-thoracic fractures or those associated with shock, while patients requiring either a thoracotomy or laparotomy require early ventilation and are more likely to experience complications [27, 28].

Cardiac injury can be deleterious; however, clinical studies looking at blunt cardiac injuries in chest and abdominal trauma report incidences significantly lower than in autopsy series. This could imply that milder forms of cardiac injury go unnoticed, or that many patients with significant cardiac injuries die in the field from cardiac or associated traumatic injury. ECG can screen for rhythm and conduction disturbances before TEE detects segmental wall abnormalities or valvular dysfunction [29]. This is consistent with our report, where presence of myocardial contusion was correlated with overall mortality.

In flail chest injuries, the flail fragment commonly causes a laceration, contusion, pneumothorax, hemothorax, or hemopneumothorax. It is worth noting that pulmonary contusion, rather than the flail, impairs considerably vital capacity after chest trauma, which shows predominantly as a restrictive defect. It is important for intensivists to understand the pathophysiology of traumatic pulmonary contusion and to implement protective approaches in mechanical ventilation to avoid further lung damage [30, 31]. In our findings, evident lung contusions were significantly correlated with overall mortality.

Traumatic pneumothorax in flail chest cases is caused by laceration of the parietal pleura by the fractured rib edges, causing leakage of air into the pleural space. Loss of the pleural negative pressure leads to lung collapse, possible respiratory failure, and, in more severe cases, shock. If recognized in a timely manner and treated appropriately, traumatic pneumothorax does not have any serious consequences. In most cases, chest tube placement is the definitive therapeutic action [32, 33]. In our review, all cases presented with pneumothorax clinically and confirmed by imaging, chest roentgenogram, computed tomography, with or without ultrasound, had chest tubes inserted in timely manner. Presence of pneumothorax and chest tubes had correlated directly with overall mortality.

Moreover, associated surgeries, pneumonia, steroids, and standard fluid therapy were significantly correlated with mortality. In fact, corticosteroids are not thought to be beneficial for pulmonary contusion management and may even be harmful. By reducing bacterial clearance, corticosteroids may increase the risk of pneumonia [34]. We noted higher mortality related to the prescription of steroids for contusion in the current report. In fact, all steroids prescribed for flail chest patients in this report were at very high doses (more than 100 mg of prednisone equivalent a day). Low-dose steroids are not recorded for evaluation and benefit identification.

It is generally known that there is no justification for giving prophylactic antibiotics to patients who have suffered traumatic chest wall injuries [34]. Administration of prophylactic antibiotics for patients with blunt and penetrating chest injuries necessitated the insertion of a chest drain was associated with a low risk of developing post-traumatic pneumonia and empyema [35]. Data on the prescription of prophylactic antibiotics for our patients revealed that they were not specifically connected to flail chest but rather to the associated variable conditions, such as preoperatively or before the placement of a chest tube in penetrating chest injuries.

Sepsis encountered in polytrauma patients, as a result of neglected open or surgical wounds, or following prolonged MV, is a frequent complication which constitutes a major concern for intensivists for its direct impact on prognosis. Proper care of open wounds and tubes, ventilatory care bundle as well as strict antimicrobial protocol implementation can be cumbersome for cases with late admission to hospitals or shifted between different departments or health care facilities [10]. In our study, sepsis was carefully monitored and addressed. However, sepsis contributed as an independent factor for mortality.

In the majority of trauma settings, the judicious administration of fluid is recommended and is especially important in the flail chest as lung contusions are so common. Although it has been shown that “fluid restriction “improves oxygenation in animal models, there is currently no convincing human evidence to support the outcome improvement. Patients should be adequately resuscitated to maintain signs of tissue perfusion without being excessively fluid-restricted. Diuretics may be beneficial in cases of volume overload [36, 37]. Concerning this, current study shows that patients with flail chest injuries benefit from restricted fluid therapy.

In our findings, patients served with regional analgesia had a significantly higher survival rate than those who were only given an intravenous fentanyl infusion. Our finding coincides with the given study, in which an infusion of bupivacaine through continuous thoracic paravertebral catheterization provided adequate pain relief and improved the respiratory condition in a patient with unilateral multiple fractured ribs. These measurements also improved patients’ conditions for four days, reducing pain and improving respiratory condition and oxygenation [38]. Similar findings were reported in a recent retrospective systematic review of 119 articles, where epidural analgesia was found to be a better pain reliever than the other modalities. However, the authors of that study stated that the quality of the available evidence is low, and therefore they rule out strong endorsements of the findings [39]. On the other hand, the efficacy of intravenous infusions of anesthesia, i.e., fentanyl and lidocaine, in comparison to either continuous epidural or paravertebral analgesia was found to be the lowest in many reported studies [40, 41]. These concomitant findings support ours that continuous regional analgesic technique—more importantly, epidural analgesia—exerts the best outcome regarding mortality. The main factor in choosing the type of regional anesthesia was the treating physician’s clinical judgment. Gender, and MV use, on the other hand, had no statistical significance on mortality.

### Limitations

Despite our best efforts to collect specific data about flail chest patients, the study has several limitations. Due to the possibility of unmeasured parameters, numerous comparisons, and a single center, our data should be taken with caution. Another problem is the absence of surgical fixation data, which has just lately gained wider acceptance. Actually, because the study was retrospective and only a few cases had been surgically repaired, it is regrettable that they were not enrolled.

Despite these limitations, our study offers a benefit because the data collection was paired and focused primarily on the flail chest.

## Conclusion

The current report recorded mortality of 19.9% between flail chest injury patients. The present data indicate that sepsis, concomitant head injury, and higher ISS are the independent risk factors for mortality when associated with flail chest injury. Mechanical ventilation support had no demonstrable impact on mortality. Considering restricted fluid management strategy and regional analgesia may help better outcome for flail chest injury patients.

## Data Availability

Data can be obtained from the corresponding authors upon reasonable request.

## Abbreviations

ICU: Intensive Care Unit
SICU: Surgical Intensive Care Unit
EICU: Emergency Intensive Care Unit
UK: The United Kingdom
PVB: Paravertebral block
ECG: Electrocardiogram
TEE: Transesophageal echocardiography
MV: Mechanical ventilation
AIS: Abbreviated injury score
ISS: Injury severity score
